# Digital marketing of online food delivery services in a social media platform before and during COVID-19 pandemic in Brazil

**DOI:** 10.1017/S1368980022002191

**Published:** 2022-10-10

**Authors:** Laís Vargas Botelho, Jade Veloso Freitas, Alex Oliveira da Camara, Iasmim Ferreira de Almeida, Thauanne de Souza Gonçalves, Paula Martins Horta, Daniela Silva Canella, Letícia de Oliveira Cardoso

**Affiliations:** 1 Sergio Arouca National School of Public Health, Oswaldo Cruz Foundation, Rio de Janeiro, Brasil. Leopoldo Bulhões St., 1480 – Manguinhos, Rio de Janeiro, RJ 21041-210, Brazil; 2 Institute of Social Medicine, Rio de Janeiro State University, Rio de Janeiro, Brazil; 3 Department of Nutrition, Federal University of Minas Gerais, Belo Horizonte, Brazil; 4 Institute of Nutrition, Rio de Janeiro State University, Rio de Janeiro, Brazil

**Keywords:** Digital food environment, Online food delivery platforms, Digital marketing, Food promotion, Ultra-processed food, Social media, COVID-19

## Abstract

**Objective:**

To describe the promotion of food and beverage and marketing strategies used by online food delivery services (OFDS) in a social media platform before and during the pandemic in Brazil.

**Design:**

Publicly available data were extracted from OFDS Instagram accounts. Posts published 6 months immediately before and after the first case of COVID-19 in Brazil were randomly sampled. Two independent authors coded the posts’ content. Food and beverage items featured in posts were classified according to the NOVA food system classification. Marketing strategies were coded according to protocols from previous studies.

**Setting:**

Top three OFDS Instagram accounts in Brazil.

**Participants:**

Posts published in the period studied (*n* 304).

**Results:**

During the pandemic, the proportion of posts featuring at least one food item decreased from 71·6 % to 40·2 %, and the proportion of ultra-processed foods decreased from 57·6 % to 27·9 %. Before the pandemic, the most widely used marketing strategies were branding elements (80·7 %), product imagery (unbranded) (48·9 %) and partnerships/sponsorship (35·2 %). While during the pandemic, branding elements (62·2 %) continued to be the most applied, but were followed by the use of videos/graphics interchange format/boomerangs (34·1 %) and corporate social responsibility (31·7 %). The most frequent COVID-19 marketing strategies were ‘social responsibility in the pandemic’ (30·5 %), ‘combatting the pandemic’ (28·0 %) and ‘accelerating digitalisation’ (20·7 %).

**Conclusions:**

OFDS advertisements on a social media platform placed less emphasis on food items, but improved the nutritional quality of foods and beverages featured in posts. A COVID-washing approach was highlighted, especially through the use of social responsibility marketing during the pandemic.

COVID-19, the infectious disease caused by SARS-COV-2, emerged in December 2019 in Wuhan, China, and quickly reached pandemic status^(1)^. The Brazilian Health Ministry confirmed the country’s first case on 26 February 2020^(2)^ and had recorded 5 million cases and 150 thousand deaths by August that year. The first wave of the disease in the country lasted from March to November 2020^(3)^ and reached its peak in August 2020^(2)^.

In order to contain the spread of the virus, Brazilian states and municipalities implemented several plans such as closing schools and public recreational areas, adopting remote work in several sectors and establishing specific rules to keep essential services functioning while interrupting non-essential ones^(4,5)^. Individual behaviours have also changed. About 70 % of adolescents and adults were practicing physical distancing between April and May 2020^(6)^, and 8·4 million Brazilians were working in home office mode in August 2020^(7)^. With part of the population spending more time at home, there was an increase in time spent using electronic devices such as computers, tablets and TVs^(8)^.

The digital food environment encompasses three factors that could influence diet-related outcomes: digital actors, digital settings where such actors operate and digital activities performed by such actors^(9)^. According to this framework, the food industry is a digital actor that performs activities such as digital marketing in digital arenas (e.g. social media). We considered digital marketing as any promotional activities undertaken through websites, social media platforms, e-mails, mobile phone texts, apps and online games targeted to individuals, groups and/or populations^(10)^.

The main digital and media marketing features used to target young people are immersive environments, active engagement, user-generated content, personalisation through ‘big data’, ubiquitous connectivity and the social graph – the complex web of relations that enables marketers to access and influence interconnected individuals^(11,12)^. In particular, social media, which consists of apps that enable content creation and sharing^(13)^, are among the most widely accessed digital marketing arenas. As the popularity of this type of platform has been growing, companies have been developing a variety of strategies to reach new audiences there, including those from the food and beverage industry^(12)^.

Previous studies have shown that digital marketing on social media is centred on ultra-processed food and contributes to the consumption of unhealthy items, as it influences product recognition, consumer beliefs and preferences, purchase intention, food choices and eating behaviours^(11,14)^. Considering the increase in the use of digital devices during physical distancing, it is plausible that the use of social media has also increased along with rising exposure to digital food marketing^(15,16)^.

The pandemic’s context has also motivated the use of online food delivery services (OFDS), explained by the shift of establishments that sell food prepared away from home to the digital environment, the use of digital technology in daily activities and the promotion of this type of service in the digital environment^(17)^. According to data collected from July to September 2020^(18)^, the percentage of internet users who purchased food or food products online in Brazil increased, especially among consumers who ordered snacks and meals on OFDS (from 15 % in 2018 to 44 % during the pandemic). During the first wave of COVID-19 in Brazil, food advertising also started to stimulate food delivery and convey messages related to physical distancing, reinforcing the idea of staying home with the family and encouraging personal interaction through digital technologies^(19)^.

Consumption of ultra-processed foods is associated with obesity and non-communicable diseases^(20)^. One study conducted in Brazil showed that the digital food environment of OFDS has prioritised ultra-processed food during the pandemic, as free delivery ads, promotional combos and discount messages in the apps were mainly targeted towards ultra-processed foods^(21)^. However, there are only a few studies^(22,23)^ on digital marketing by OFDS in other digital environments such as video-sharing websites and social media platforms, and they were conducted in high-income countries.

Considering the potential of digital food marketing to influence food and beverage consumption and the growth of OFDS use during the pandemic, it is important to understand how these companies advertise their services on a popular social media platform. This study, therefore, aims to analyse the content of marketing strategies and the promotion of food and beverages in the top three OFDS Instagram accounts in Brazil, comparing their behaviour before and during the first wave of the COVID-19 pandemic in the country.

## Methods

### Sampling and data extraction

We selected verified publicly available Instagram accounts from the top three OFDS operating in Brazil: iFood®, UberEats® and Rappi®. They were chosen because they were the food delivery market leaders in the country^(24)^. Instagram was chosen due to its widespread use among young Brazilian adults^(25)^, who also are the main population segment among OFDS users^(26)^.

We used the free version of a cloud-based data extraction software to automatically scrape data from posts published in the period between 26 August 2019 and 26 August 2020 – 6 months before and after the first case of COVID-19 was recorded in the country, respectively. Therefore, it included the first wave of the pandemic in Brazil^(2)^. The information extracted from Instagram accounts were the current number of followers, number of posts during the period studied, number of posts since the account’s creation, date of each post, link to access the posts, post type (photo or video), number of likes and comments and number of views (exclusive for video posts).

### Content coding protocol

Two independent authors performed a three-phase content coding protocol adapted from previous work by Jia et al.^(22)^. Discrepancies were discussed and solved by a third encoder.

In Phase 1, we coded the nutritional quality of foods or beverages for the posts in which these elements were present. The classification was made according to the Dietary Guidelines for the Brazilian Population^(27)^. It is based on the NOVA classification, a food categorisation system based on the extent and purpose of the foods´ industrial processing^(28)^.

We used NOVA to classify dishes and culinary preparations based on a decision tree^(29)^ that categorises food, preparations and beverages into three groups: (1) unprocessed or minimally processed foods or hand-prepared dishes based on these foods; (2) processed foods or (3) ultra-processed foods (online Supplementary Table S1). We examined the number of foods or beverages presented in the post and the number of items in each group. The authors who coded the nutritional quality are nutritionists.

In Phase 2, we coded marketing strategies used by OFDS on Instagram posts, considering coding frameworks developed in a former study on social media junk food promotion^(30)^. We also assessed whether each post had informative content, original content and/or health claims (online Supplementary Table S1).

In Phase 3, we coded posts published during the first wave of the pandemic in Brazil in four thematic categories as proposed by Jia et al.^(22)^: (i) appropriating frontline workers; (ii) combatting the pandemic via promotions; (iii) selling social distancing and (iv) accelerating digitalisation (online Supplementary Table S1).

In Phases 2 and 3, posts could be coded into multiple categories if more than one marketing strategy was present. The classification was performed by viewing the posts and reading their captions. If a strategy could not be clearly coded in one of the pre-established categories, each encoder described the strategy to discuss with the third encoder whether it was necessary to build a new category. Thus, in Phase 2, a new category emerged and was called ‘meme’, referring to the use of any type of content that can viralise on the internet and that is modifiable during peer-to-peer transmission. In Phase 3, we added the categories ‘Support to delivery drivers’, ‘Support to restaurants’ and ‘Social responsibility during the pandemic’ – divided into ‘Corporate social responsibility’ and ‘Individual social responsibility’ (online Supplementary Table S1).

In order to better understand the marketing messages related to COVID-19 in the specific Brazilian context, we conducted case studies illustrating each Phase 3 category.

### Statistical analysis

The total number of posts in the period studied (*n* 304) was considered to describe the accounts´ characteristics. For the three-phase content coding, we took a simple random sample of the posts, considering a 95 % level of significance, a 5 % margin of error and a 50 % heterogeneity (*n* 170).

We performed a descriptive analysis of the posts published before and during the COVID-19 pandemic. We calculated some interaction proxies: the average number (minimum – maximum) of likes and comments per post and of views per video post and the relationship between the number of likes/comments/views and the number of followers. We also calculated absolute and relative frequencies of each NOVA food group, general marketing strategy and COVID-19 marketing strategy.

## Results

### Online food delivery services Instagram accounts characteristics

By the time of the present study’s data scrapping (June 2021), the three Brazilian Instagram accounts together had 1·84 million followers and a total of 1468 publications since their inception. The iFood® account had more followers (1·1 million, 59·78 %) and posts (852, 58·44 %), followed by Rappi® (460 thousand followers, 25·00 %; 523 posts, 35·87 %) and UberEats® (280 thousand followers, 15·22 %; 93 posts, 6·38 %). iFood’s first post was published in 2015/01, UberEats’ in 2019/11 and Rappi’s in 2017/07.

In the 6 months before the pandemic (26 August 2019 to 25 February 2020), 11·10 % (163/1468) of all publications were posted. During the pandemic, that is, in the first 6 months of the first wave in Brazil (February 26, 2020 to August 26, 2020), this percentage was lower and equal to 9·60 % (141/1468). Specifically regarding videos, the number of posts with this type of media before the pandemic was lower than during it (42 *v*. 51). Average likes (1113 *v*. 1412), comments (306 *v*. 617) and video views increased (13 045 *v*. 98 511) during the pandemic, but active interaction (likes and comments) was low (<1 %) relative to the number of total account followers. Passive interaction (video viewing), in turn, reached a higher percentage of followers, especially during the pandemic (1·70 % *v*. 10·00 %) and in the iFood® account (1·30 % *v*. 15·60 %) – even though this company had posted the same number of videos (*n* 27) in both periods (Table 1).

**Table 1:** Account characteristics on a social media platform of online food delivery services before and during COVID-19 pandemic, Brazil

Online food delivery services’ Instagram account characteristics	Total (*n* 304)				Before the pandemic (*n* 163)				During the pandemic (*n* 141)			
	Overall	iFood®	UberEats®	Rappi®	Overall	iFood®	UberEats®	Rappi®	Overall	iFood®	UberEats®	Rappi®
Total posts during study period, *n*	304	191	55	58	163	113	17	33	141	78	38	25
Average likes per post	1252	1564	523	914	1113	1162	716	1150	1412	2146	436	604
Range of likes per post	172–44 918	369–44 918	172–1514	290–4962	240–4962	369–4374	240–1514	290–4962	172–44 918	501–44 918	172–1381	312–1118
Ratio between likes/number of followers, %	0·16	0·14	0·19	0·20	0·15	0·11	0·26	0·25	0·17	0·20	0·16	0·13
Average comments per post	451	325	282	1024	306	241	188	592	617	448	324	1595
Range of comments per post	49–4068	49–3047	102–1742	202–4068	49–1954	49–1954	111–332	202–1433	56–4068	56–3047	102–1742	533–4068
Ratio between comments/number of followers, %	0·8	0·03	0·10	0·22	0·05	0·02	0·07	0·13	0·12	0·04	0·12	0·35
Total videos during study period, *n*	93	54	8	31	42	27	1	14	51	27	7	17
Average views per video	59 914	93 093	5681	16 113	13 045	14 102	5336	11 558	98 511	172 083	5730	19 864
Range of views per video	2916–1 944 197	6834–1 944 197	2916–13 008	4750–133 836	5012–25 499	7146–22 396	–	5012–25 499	2916–1 944 197	6834–1 944 197	2916–13 008	4750–133 836
Ratio between views/number of followers, %	6·30	8·50	2·00	3·50	1·70	1·30	1·90	2·50	10·00	15·6	2·10	4·30

### Nutritional quality of featured foods

Among publications posted before the pandemic, 71·59 % (63/88) included images of at least one food or beverage. Overall, 139 food items were identified and fifty-three (38·13 %) were unprocessed or minimally processed foods or hand-prepared dishes based on these foods, six of which (4·32 %) were processed foods, and eighty of which (57·55 %) were ultra-processed foods. Furthermore, ultra-processed foods were predominant in 58·70 % (37/63) of the posts featuring food items. Most of the foods and beverages identified appeared in the iFood® account (96/139), and the posts from this company also showed a higher percentage of ultra-processed foods (63/96, 65·62 %) compared with UberEats® (9/21, 42·86 %) and Rappi® (8/22, 36·36 %).

During the pandemic, posts featuring foods or beverages decreased to 33 (40·24 %), and the number of food items shown in the posts decreased to 86. This was also seen individually in the iFood and Rappi accounts. Regarding UberEats®, although the number of posts featuring food items had increased, there was a proportional reduction in the number of foods and beverages shown. In the iFood® account, posts showing food items decreased by about 3·5 times, and the number of items decreased approximately threefold. The percentage of unprocessed or minimally processed foods or hand-prepared dishes based on these foods increased to 69·76 % (60/86) and ultra-processed foods decreased to 27·91 % (24/86). Therefore, posts in which ultra-processed foods or beverages were predominant also decreased to only 11 (33·30 %). This trend was observed in the accounts of all companies individually (Table 2).

**Table 2:** Nutritional quality of food items featured in online food delivery services’ posts on a social media platform before and during COVID-19 pandemic, Brazil

Nutritional quality of food items featured in Online Food Delivery Services Instagram posts	Overall (*n* 170)				iFood® (*n* 101)				UberEats® (*n* 36)				Rappi® (*n* 33)			
	Before		During		Before		During		Before		During		Before		During	
	*n*	%	*n*	%	*n*	%	*n*	%	*n*	%	*n*	%	*n*	%	*n*	%
Total posts during study period, *n*	88		82		56		45		11		25		21		12	
Posts featuring at least one food item	63	71·59	33	40·24	46	82·10	13	28·90	8	72·70	17	68·00	9	42·90	3	25·00
Total number of food items, *n*	139		86		96		33		21		33		22		20	
Group 1*	53	38·13	60	69·76	33	34·38	18	54·54	9	42·86	23	69·69	11	50·00	19	95·00
Group 2†	6	4·32	2	2·33	0	0·00	1	3·03	3	14·28	1	3·03	3	13·64	0	0·00
Group 3‡	80	57·55	24	27·91	63	65·62	14	42·43	9	42·86	9	27·28	8	36·36	1	5·00
Posts displaying > 50 % ultra-processed food items	37	58·70	11	33·30	29	63·00	8	61·50	5	62·50	3	17·60	3	33·30	0	0·00

* Unprocessed or minimally processed foods or hand-prepared dishes based on these foods.

† Processed foods.

‡ Ultraprocessed foods.

### Marketing strategies

Before the pandemic, fourteen out of the fifteen marketing strategies assessed were used by the OFDS, ranging from seven to thirteen strategies applied by each company. The most frequently used were branding elements (80·70 %), product imagery (unbranded) (48·90 %) sponsorships/partnerships (35·20 %), use of videos/graphics interchange format/boomerangs (28·40 %) and links (to additional content, external pages from partner restaurants or from the platforms themselves) (31·80 %). Only one post on corporate social responsibility was published before the pandemic. The new category ‘meme’ was applied in 23·90 % of the posts published before the pandemic (Table 3).

**Table 3:** Marketing strategies used in online food delivery services’ posts on a social media platform before and during COVID-19 pandemic, Brazil

Marketing strategies used in Online Food Delivery Services Instagram posts	Overall (*n* 170)				iFood® (*n* 101)				UberEats® (*n* 36)				Rappi® (*n* 33)			
	Before		During		Before		During		Before		During		Before		During	
	*n*	%	*n*	%	*n*	%	*n*	%	*n*	%	*n*	%	*n*	%	*n*	%
Total posts during study period, *n*	88		82		56		45		11		25		21		12	
Informational posts	28	31·80	23	28·00	22	39·30	14	31·10	1	9·10	3	12·00	5	23·80	6	50·00
Original posts	85	96·60	80	97·60	56	100	45	100	8	72·70	23	92·00	21	100	12	100
Health claims	1	1·10	2	2·40	1	1·80	1	2·20	0	0·00	1	4·00	0	0·00	0	0·00
Marketing strategies used (*n* 15)	14		10		11		10		7		10		13		8	
Corporate social responsibility	1	1·10	26	31·70	0	0·00	14	31·10	0	0·00	8	32·00	1	4·80	4	33·30
Celebrities	16	18·18	9	10·97	14	25·00	7	15·60	0	0·00	1	4·00	2	9·50	1	8·30
Sportspeople	1	1·10	0	0·00	0	0·00	0	0·00	0	0·00	0	0·00	1	4·80	0	0·00
Children’s characters	1	1·10	0	0·00	0	0·00	0	0·00	0	0·00	0	0·00	1	4·80	0	0·00
Branded characters	1	1·10	0	0·00	1	1·80	0	0·00	0	0·00	0	0·00	0	0·00	0	0·00
Special price promotions	16	18·20	11	13·40	15	26·8	10	22·20	0	0·00	1	4·00	1	4·80	0	0·00
Vouchers	6	6·80	0	0·00	5	8·90	0	0·00	0	0·00	0	0·00	1	4·80	0	0·00
Competitions	0	0·00	0	0·00	0	0·00	0	0·00	0	0·00	0	0·00	0	0·00	0	0·00
Engagement	9	10·20	14	17·10	5	8·90	4	8·90	2	18·20	8	32·00	2	9·50	2	16·70
Memes	21	23·90	2	2·40	19	33·90	2	4·40	1	9·10	0	0·00	1	4·80	0	0·00
Sponsorships/partnerships	31	35·20	8	9·80	25	44·60	4	8·90	1	9·10	3	12·00	5	23·80	1	8·30
Videos/GIF/boomerangs	25	28·40	28	34·10	14	25·00	17	37·80	1	9·10	5	20·00	10	47·60	6	50·00
Links	28	31·80	22	26·80	23	41·10	19	42·20	1	9·10	2	8·00	4	19·00	1	8·30
Branding elements	71	80·70	51	62·20	51	91·10	37	82·20	2	18·20	8	32·00	18	85·70	6	50·00
Product imagery (unbranded)	43	48·90	23	28·00	32	57·10	8	17·80	7	63·60	12	48·00	4	19·00	3	25·00

GIF, graphics interchange format.

During the pandemic, ten different strategies were applied, and we observed a reduction in the use of branding elements (62·20 %), product imagery (unbranded) (28·00 %) and links (26·80 %), but these remained among the most widely used strategies along with the use of videos/graphics interchange format/boomerangs (34·10 %) and corporate social responsibility (31·70 %) (Table 3).

Only Uber Eats® adopted the practice of ‘regramming’ customers’ content, before or during the pandemic. The entire content produced by the other two brands was original in both time periods (see definitions in online Supplementary Table S1). We observed health claims in only one post before the pandemic and in two posts during the pandemic. The prevalence of informative posts did not differ before and during the pandemic (31·80 % *v*. 28·00 %) (Table 3). However, before the pandemic, informative content was primarily about the physical location of the partner restaurants´ food outlets, while during the pandemic it mainly included new features added to the apps due to the pandemic.

### Marketing strategies related to COVID-19

Among publications posted during the first wave of COVID-19, 53·66 % referred directly or indirectly to the context of the pandemic. The iFood® account was the most active during the period, publishing forty-five posts, 46·67 % of which mentioned the pandemic. Overall, UberEats® published fewer posts. However, this brand made proportionally more references to the pandemic. Meanwhile, only three posts in the Rappi® account mentioned the pandemic (Table 4).

**Table 4:** Content analysis of COVID-19 marketing strategies in online food delivery services’ posts on a social media platform during COVID-19 pandemic (*n* 82), Brazil

COVID-19-related marketing strategies from Online Food Delivery Services Instagram posts	Overall		iFood®		UberEats®		Rappi®	
	*n*	%	*n*	%	*n*	%	*n*	%
Total posts during the period, *n*	82		45		25		12	
Posts related to COVID-19	44	53·66	21	46·67	20	80·00	3	25·00
Combatting the pandemic via promotions	23	28·00	10	22·22	11	44·00	2	16·67
Accelerating digitalisation	17	20·70	7	15·56	9	36·00	1	8·33
Support for delivery drivers	12	14·60	10	22·20	2	8·00	0	0·00
Support for restaurants	12	14·60	5	11·10	7	28·00	0	0·00
Social responsibility during the pandemic	25	30·50	14	31·11	8	32·00	3	25·00
Corporate social responsibility	19	76·00	14	100·00	3	37·50	2	66·67
Individual social responsibility	10	40·00	1	7·14	8	100·00	1	33·33

The categories ‘appropriating frontline workers’ and ‘selling social distancing’ are not shown because they were not identified in the study.

Our analysis of marketing strategies related to COVID-19 showed that the predefined analytical categories ‘appropriating frontline workers’ and ‘selling social distancing’ were not used in any post. However, we defined three important non-mutually exclusive categories: ‘social responsibility in the pandemic’ (30·50 %), the most frequently used, ‘support for delivery drivers’ (14·60 %) and ‘support for restaurants’ (14·6 %). ‘Social responsibility in the pandemic’ had two sub-analytical categories: ‘corporate social responsibility’ (76·00 %) and ‘individual social responsibility’ (40·00 %). ‘Combating the pandemic via promotions’ (28·00 %) and ‘accelerating digitalisation’ (20·70 %) were the second and third most widely used strategies (Table 4).

### Case studies - COVID-19 marketing strategies

We present some case studies below illustrating each strategy related to the COVID-19 context (see posts used as examples in the Figures in Supplemental Table S2).

#### Social responsibility in the pandemic

Before the pandemic, ‘corporate social responsibility’ was an uncommon practice by OFDS. However, such actions became frequent during the first wave in Brazil. We also identified the emerging practice of marketing ‘individual social responsibility’. Together, the two categories were generically called ‘social responsibility during the pandemic’ and were the most frequently applied during this period.

- *Corporate social responsibility*: OFDS targeted social responsibility actions to the various parties affected by the pandemic. Online Supplementary Figure 1 shows an example in which iFood® announces the creation of a protective fund for delivery drivers as well as actions to prevent coronavirus infection among them. Online Supplementary Figure 2 shows Uber Eats® calling its customers to donate food and hygiene items to a Brazilian non-governmental organisation and encouraging on-going donations via its own mobile app.
- *Individual social responsibility*: Customers were also encouraged to act on behalf of the various entities affected by the pandemic. UberEats® encouraged its customers to tip delivery drivers (online Supplementary Fig. 3) and announced a new app feature that allowed customers to donate to establishments to help its professionals (online Supplementary Fig. 4). Also, concerning community support, iFood® encouraged platform users to donate money to be converted into food baskets delivered by a non-governmental organisation to families throughout Brazil (online Supplementary Fig. 5).

#### Combatting the pandemic via promotions

Online Supplementary Figure 3 illustrates how OFDS linked the use of their services to health recommendations during the pandemic: UberEats® underscored its commitment to supporting the community and helping to keep the cities where it operated safe by granting a free delivery fee. The company also highlighted the possibility of contactless delivery. iFood® also encouraged the option of contactless delivery (online Supplementary Fig. 6).

#### Accelerating digitalisation

Online contact became the safest possible means of interaction between people during the pandemic, affecting relationships with family and friends. OFDS grasped the context of accelerating digitalisation with increased virtual interaction to encourage consumers to use the platform. Rappi®, for example, encouraged customers to protect their grandparents by buying groceries to be delivered to their homes via the app (online Supplementary Fig. 7). Uber Eats® claimed that its customers were not alone because they were all united by food (online Supplementary Fig. 8). iFood® encouraged users to buy a meal for their mothers in celebration of Mother’s Day (online Supplementary Fig. 9) and to make Easter purchases for their families as a way to stay close to their loved ones (online Supplementary Fig. 10).

#### Support for delivery drivers

The coding *‘support for delivery drivers’* included (individual or corporate) actions in ‘social responsibility during the pandemic’ targeted to delivery drivers, but also posts expressing appreciation of these workers. For example, Uber Eats® encouraged its platform users to leave a message of thanks for delivery drivers (online Supplementary Fig. 11). iFood® posted real stories of its drivers to thank them for their work during the pandemic (online Supplementary Fig. 12).

#### Support for restaurants

Likewise, the category *‘support for restaurants’* included actions of individual or corporate ‘social responsibility during the pandemic’ but also other expressions of support to restaurants. Uber Eats® encouraged customers to choose neighbourhood establishments to support community development (online Supplementary Fig. 13) and provided space for customers to cite local restaurants that were open for delivery (online Supplementary Fig. 14). iFood® also posted stories of restaurant owners to value them and help advertise their business (online Supplementary Fig. 15).

## Discussion

This study investigated the marketing strategies and promotion of food and beverages on Instagram accounts of the top three OFDS in Brazil, comparing the period prior to the pandemic with the first wave of COVID-19 in the country. The presence of food in OFDS advertising has decreased, the nutritional quality of food items displayed in posts has improved and the use of marketing strategies changed, as the social responsibility marketing was emphasised during the pandemic.

The nutritional quality of the foods and beverages displayed during the pandemic has improved. Before, the proportion of posts featuring ultra-processed foods and beverages was close to that found by Jia et al. (58·3 %)^(22)^. But during the first wave in Brazil, unprocessed or minimally processed foods or hand-prepared dishes based on these foods became predominant. In Australia, UK and USA, the scenario was different and products with the worst nutritional quality prevailed in OFDS Instagram accounts^(22)^ during the pandemic. These differences may be partly an expression of the different dietary patterns in the countries analysed: while ultra-processed foods comprise 21·5 % of energy intake in Brazil, the proportions reach 42·0 %, 56·8 % and 57·5 % in Australia, UK and USA, respectively^(14)^. However, other factors may explain these changes in advertising. The online study NutriNet-Brasil^(31)^ found a modest increase in the frequency of consumption of healthy eating markers (vegetables, fruits and beans) and stability in the consumption of ultra-processed foods during the first months of the pandemic. This cohort consisted mostly of highly educated people living in the most economically favoured regions of Brazil. Food delivery app users in Brazil also belong to these socio-economic strata and regions^(26)^. Furthermore, during the pandemic, social discourses began to reinforce the importance of maintaining healthy lifestyles^(32)^. Thus, OFDS may have emphasised the image that the delivery service could be used to purchase healthy food because its main target audience was more concerned about improving nutrition to increase immune defenses against COVID-19^(33)^.

Although ultra-processed food promotion has been reduced on Instagram during the pandemic, evidence shows that availability and food advertising of unhealthy foods exceed that of healthy foods in the digital environment of the OFDS platforms^(21,34,35)^, including in Brazil^(22)^. Additionally, iFood reported that sfihas, hamburgers (hand-prepared or fast-food type), pizzas, sodas, sandwiches and wraps were among the most frequently ordered items during the pandemic^(36)^. Also, a web survey conducted with highly educated adults in Greater Metropolitan Rio de Janeiro, Brazil, found that fast food, homemade hamburgers and pizzas, sodas, oriental food and Brazilian meals remained among the top five ordered food items by OFDS users, although the proportion of fast foods and sodas orders decreased during the pandemic^(37)^. This set of information demonstrates inconsistency between OFDS Instagram advertising and the impact of delivery on food consumption during the pandemic.

Regarding the presence of marketing strategies in the posts, the three OFDS mainly used branding elements and product imagery (unbranded) strategies in both periods. During the pandemic, the use of these approaches decreased compared with the previous period because considerable space was shifted to social responsibility messages. As happened in Australia, UK and USA^(22)^, OFDS could have taken advantage of their growing popularity and use during the health crisis in Brazil^(36)^ to employ branding elements such as colours, logos and symbols to consolidate identity by improving recognition and recall, which has a positive impact on purchase intention^(14)^. Contrarily, the number of posts decreased (except specifically from UberEats®), and companies focused on showing awareness and engagement in facing the consequences of the pandemic to improve their corporate image.

The tendency to encompass issues related to the pandemic was also observed in other aspects analysed. For instance, during the pandemic, links to extra content consisted mostly of preventive measures and actions taken by OFDS towards the coronavirus and informational posts focused on new possibilities for buying groceries, requesting contactless delivery and making donations through the app. Also, before the pandemic, more than 20 % of posts used memes to generate engagement through relaxed and familiar language to social media users. However, the tone of marketing messages has changed, and only two posts used this strategy during the health crisis in Brazil.

With respect to pandemic-related marketing strategies, the most commonly used in high-income countries was ‘combatting the pandemic via promotions’^(22)^. While in Brazil, this was the second most widely used, following ‘social responsibility during the pandemic’. It is worrisome because although young adults realise that corporate social responsibility digital marketing is designed to promote a product, their interest in it is highly affected by these actions^(38)^. Further investigation showed that these responsibility messages during the pandemic in Brazil referred not only to ‘corporate social responsibility’ but also to ‘individual social responsibility’, encompassing a prominent discourse evoking individual actions of social responsibility by customers. According to Abid, Abid-Dupont & Moulins^(39)^, ‘brands can encourage consumer commitment through their environmental and philanthropic engagements by conveying values with which consumers can identify to foster brand identification’. The frequent use of social responsibility discourses was probably motivated by the underlying economic and social crisis, which resulted in increased unemployment and food insecurity rates and became an agenda for marketing in different areas^(40)^.

This type of pro-cause marketing was extensively practiced by the food industries during the pandemic too^(40–42)^. It is called COVID-washing^(41,42)^ and can be defined as the appropriation of the adverse context of the pandemic by companies to show themselves empathetic and proactive in relation to the health crisis, aiming to improve their image and create positive associations with their brands^(40–42)^. COVID-washing has the potential to blur the perception of the negative impact of harmful products on public health and increase consumers´ trust, perception of quality and brand loyalty^(39,41)^. Thus, the highlight of healthy foods and beverages on OFDS Instagram accounts during the pandemic in Brazil can be considered part of this marketing strategy to reshape brand image since it is inconsistent with the greater availability of ultra-processed foods in their applications.

Additionally, OFDS used discourses about supporting delivery drivers and restaurants during the pandemic. Such findings corroborate those by Jia et al.^(22)^, who commented that OFDS served messages and hashtags to make users feel they could support local restaurants and delivery drivers. However, these messages appear to have been more widely used in the Brazilian context, probably to appease the social mobilisation generated by a strike by delivery drivers demanding better working conditions and the end of restrictions and punishments by the algorithms^(43)^. Brazilian society engaged with this cause because, during the pandemic, the population recognised that this class of workers provided essential services and was indispensable for food outlets to continue functioning. Despite this, OFDS drivers are not employees of the companies, as they are subject to a new kind of relationship called a ‘partnership’ by the companies. Therefore, they assume the work’s risks and costs, while the OFDS only provides the infrastructure for their ‘partners’ to conduct the activities^(44)^. At the same time, small restaurant owners’ complaints about OFDS charging abusive fees and lowering the visibility of their business on the platform resonated during the COVID-19 crisis due to the economic vulnerability of small and medium-sized businesses^(45)^.

These findings highlight the relevance of empirical research investigating how digital marketing techniques can affect consumers’ purchase intention. It is important to continually monitor the food delivery industry’s advertising on social media to identify how they adapt their messages to transformations in the social context to better persuade the public, as we observed about COVID-washing. Future research should also focus on investigating whether OFDS’ food promotion and marketing strategies have undergone any lasting post-pandemic changes and elucidate how exposure to persuasive content on the digital landscape can impact individual eating practices. Currently, marketing legislation is not fully adapted and extended to the digital food environment of social media^(46)^, so this set of evidence could support public policies to regulate industry’s acting in the digital environment and to promote both overall digital literacy and health and nutrition literacy.

Policymakers should include brand marketing in social media within the scope of advertising regulations to prevent companies from promoting unhealthy foods using their branding elements and generating loyalty^(40,41)^ as well as protect society against social responsibility actions. Although such actions may seem harmless, they contribute to maintaining the status quo. For example, OFDS demonstrated concern about the consequences of the pandemic and announced actions to support delivery drivers and small restaurants, but their complaints were not addressed, neither was there any substantial change in the asymmetric relationships between the parties involved in this business model. Furthermore, the highlight of healthy foods and beverages on Instagram during the pandemic can also be considered merely part of the marketing strategy to reshape brands’ image since it is known that most of the food available and sold on their platforms is associated with weight gain and chronic diseases^(20)^.

### Strengths and limitations

The present research adds to the literature on the digital food environment, as it replicated a social media monitoring methodology previously applied in three high-income countries^(22)^ to assess the promotion of unhealthy foods and the marketing strategies employed by OFDS on an important social media platform in a middle-income context, and especially by considering the advent of the COVID-19 pandemic.

A methodological strength of this study concerns the comparison between a pre-pandemic moment and the first wave of COVID-19, the period in which there was greater adherence to physical distancing by the Brazilian population. It allowed mapping strategic changes motivated by the pandemic context. Furthermore, we analysed a continuous period to guarantee that observed differences between the periods would truly have been caused by the emergence of COVID-19. Additionally, we assessed foods and beverages based on the NOVA classification, a system that considers the extent and purpose of food processing and that guides public policies on food and nutrition in the country^(27)^.

Our main limitation is the analysis restricted to the feed from only one social media platform. It is possible that the OFDS adopt different strategies in different social media platforms, and that there are differences in paid advertising. However, Instagram is a relevant platform for this research because its audience consists predominantly of people from the same prevailing age group as among food delivery users in Brazil. Moreover, the results obtained refer to the first 6 months of the pandemic in the country and may not represent later moments in which commercial establishments were gradually reopening. Lastly, it was not possible to recover the number of followers of the accounts in the period whose posts were evaluated.

## Conclusions

In Brazil, OFDS intensely practiced COVID-washing on a popular social media platform during the first wave of the pandemic by featuring mostly healthy foods and showing concern and commitment during the health, social and economic crisis.

## Supplementary material

For supplementary material accompanying this paper visit https://doi.org/ 10.1017/S1368980022002191. click here to view supplementary material

## References

[1] World Health Organization (2020) Listings of WHO’s Response to COVID-19. https://www.who.int/news/item/29-06-2020-covidtimeline (accessed July 2021).

[2] Fundação Oswaldo Cruz (2021) A balance of the pandemic in 2020: COVID-19 observatory bulletin. https://agencia.fiocruz.br/sites/agencia.fiocruz.br/files/u34/boletim_covid_6meses.pdf (accessed July 2021).

[3] Moura EC , Silva EN , Sanchez MN et al. (2021) Timely availability of public data for health management: COVID-19 wave’s analysis. https://preprints.scielo.org/index.php/scielo/preprint/view/2316/version/2454 (accessed August 2022).

[4] PGE-RJ (2020) Provides for Measures to Deal with the Spread of the New Coronavirus (COVID-19), as a Result of the Health Emergency Situation, and Other Measures. Official Gazette of the State of Rio de Janeiro. Decree nº 47,102 of June 1, 2020 (Rio de Janeiro). https://pge.rj.gov.br/comum/code/MostrarArquivo.php?C=MTEwMzg%2C (accessed July 2021).

[5] Portal do Governo de São Paulo (2020) Decrees of the Government of São Paulo with measures to prevent and combat the new coronavirus. https://www.saopaulo.sp.gov.br/spnoticias/decretos-do-governo-de-sp-com-medidas-de-prevencao-e-combate-ao-novo-coronavirus/ (accessed July 2021).

[6] Szwarcwald CL , Souza Júnior PRB , Malta DC et al. (2020) Adherence to physical contact restriction measures and the spread of COVID-19 in Brazil. Epidemiol Serv Saúde 29, e2020432. 33175010 10.1590/S1679-49742020000500018

[7] The Brazilian Institute of Geography and Statistics (IBGE) (2020) National Survey by Sample of Households (PNAD) COVID-19: Monthly Result, August 2020. https://biblioteca.ibge.gov.br/visualizacao/livros/liv101755.pdf (accessed July 2021).

[8] Malta DC , Szwarcwald CL , Barros MB et al. (2020) The COVID-19 pandemic and changes in adult Brazilian lifestyles: a cross-sectional study, 2020. Epidemiol Serv Saúde 29, e2020407. 32997069 10.1590/S1679-49742020000400026

[9] Granheim SI , Løvhaug AL , Terragni L et al. (2022) Mapping the digital food environment: a systematic scoping review. Obes Rev 23, e13356. 34519396 10.1111/obr.13356

[10] Kelly B , Vandevijvere S , Freeman B et al. (2015) New media but same old tricks: food marketing to children in the digital age. Curr Obes Rep 4, 37–45. 26627088 10.1007/s13679-014-0128-5

[11] Buchanan L , Kelly B , Yeatman H et al. (2018) The effects of digital marketing of unhealthy commodities on young people: a systematic review. Nutrients 10, 148. 29382140 10.3390/nu10020148 PMC5852724

[12] Montgomery KC , Chester J , Grier SA et al. (2012) The new threat of digital marketing. Pediatr Clin N Am 59, 659–675. 10.1016/j.pcl.2012.03.022 22643172

[13] Kraak V , Zhou M & Patiño SRG (2020) Digital marketing to young people: consequences for the health and diets of future generations. In Nutrition in a Digital World. [ DCC Delmue , SIO Granheim & S Oenema , editors]. Rome: United Nations System Standing Committee on Nutrition.

[14] Boyland E , Thivel D , Mazur A et al. (2020) Digital food marketing to young people: a substantial public health challenge. Ann Nutr Metab 76, 6–9. 10.1159/000506413 32101856

[15] Scarmozzino F & Visioli F (2020) Covid-19 and the subsequent lockdown modified dietary habits of almost half the population in an Italian sample. Foods 9, 675. 32466106 10.3390/foods9050675 PMC7278864

[16] Sheth J (2020) Impact of COVID-19 on consumer behavior: will the old habits return or die? J Bus Res 117, 280–283. 32536735 10.1016/j.jbusres.2020.05.059 PMC7269931

[17] Botelho LV , Cardoso LO & Canella DS (2020) COVID-19 and the digital food environment in Brazil: reflections on the pandemic’s influence on the use of food delivery apps. Cad Saúde Pública 36, e00148020. 33237202 10.1590/0102-311X00148020

[18] Brazilian Internet Steering Committee (2021) COVID-19 — ICT PANEL Web Survey on the Use of Internet in Brazil During the New Coronavirus Pandemic. https://cetic.br/media/docs/publicacoes/2/20210426095323/painel_tic_covid19_livro_eletronico.pdf (accessed July 2021).

[19] Rodrigues MB , Matos JP & Horta PM (2021) The COVID-19 pandemic and its implications for the food information environment in Brazil. Public Health Nutr 24, 321–326. 33222707 10.1017/S1368980020004747 PMC7737163

[20] Pagliai G , Dinu M , Madarena MP et al. (2021) Consumption of ultra-processed foods and health status: a systematic review and meta-analysis. Br J Nutr 125, 308–318. 32792031 10.1017/S0007114520002688 PMC7844609

[21] Horta PM , Matos JP & Mendes LL (2021) Digital food environment during the coronavirus disease 2019 (COVID-19) pandemic in Brazil: an analysis of food advertising in an online food delivery platform. Br J Nutr 126, 767–772. 33208203 10.1017/S0007114520004560 PMC7737114

[22] Jia SS , Raeside R , Redfern J et al. (2021) #SupportLocal: how online food delivery services leveraged the COVID-19 pandemic to promote food and beverages on Instagram. Public Health Nutr 24, 4812–4822. 34247686 10.1017/S1368980021002731 PMC8280395

[23] Martino F , Brooks R , Browne J et al. (2021) The nature and extent of online marketing by big food and big alcohol during the COVID-19 pandemic in Australia: content analysis study. JMIR Public Health Surveill 7, e25202. 33709935 10.2196/25202 PMC7958974

[24] CVA Solutions (2020) iFood, Rappi, Uber Eats: which delivery is the most appreciated by customers? https://www.cvasolutions.com/pt/ifood-rappi-uber-eats-qual-e-o-delivery-mais-bem-visto-pelos-clientes/ (accessed July 2021).

[25] Datareportal (2021) Digital 2021: Brazil. https://datareportal.com/reports/digital-2021-brazil (accessed July 2021).

[26] Instituto Qualibest (2019) Consumption of food delivery by apps. https://www.institutoqualibest.com/download/uso-de-apps-de-delivery-de-comida/ (accessed July 2021).

[27] Brazil, Ministry of Health of Brazil & Secretaria of Health Care & Primary Health Care Department (2014) Dietary Guidelines for the Brazilian Population. Brasilia: Ministry of Health of Brazil.

[28] Monteiro CA , Cannon G , Levy R et al. (2016) NOVA. The star shines bright. World Nutr 7, 28–38.

[29] Louzada MLC , Baraldi LG , Steele EM et al. (2015) Consumption of ultra-processed foods and obesity in Brazilian adolescents and adults. Prev Med 81, 9–15. 26231112 10.1016/j.ypmed.2015.07.018

[30] Vassallo AJ , Kelly B , Zhang L et al. (2018) Junk food marketing on Instagram: content analysis. JMIR Public Health Surveill 4, e9594. 10.2196/publichealth.9594 PMC6008515 29871854

[31] Steele EM , Rauber F , Costa CDS et al. (2020) Dietary changes in the NutriNet Brasil cohort during the COVID-19 pandemic. Rev Saude Publica 54, 91. 32901755 10.11606/s1518-8787.2020054002950 PMC7454165

[32] Carvajal-Miranda C , Mañas-Viniegra L & Liang L (2020) Online discourse in the context of COVID-19, the first health crisis in China after the advent of mobile social media: a content analysis of China’s Weibo and Baidu. Soc Sci 9, 167.

[33] Gaspar MC , Ruiz M , Begueria A et al. (2020) Eating in times of confinement: eating management, discipline and pleasure. Perifèria 25, 63–73.

[34] Partridge SR , Gibson AA , Roy R et al. (2020) Junk food on demand: a cross-sectional analysis of the nutritional quality of popular online food delivery outlets in Australia and New Zealand. Nutrients 12, 3107. 33053705 10.3390/nu12103107 PMC7601596

[35] Poelman MP , Thornton L & Zenk SN (2020) A cross-sectional comparison of meal delivery options in three international cities. Eur J Clin Nutr 74, 1465–1473. 32332863 10.1038/s41430-020-0630-7

[36] Exame (2020) This is the most frequently ordered item on iFood throughout Brazil. https://exame.com/negocios/esse-e-o-item-mais-pedido-no-ifood-em-todo-o-brasil/ (accessed July 2021).

[37] Botelho LV (2021) Digital food environment: a descriptive study on the use of online food delivery applications by residents of the metropolitan region of Rio de Janeiro. Master’s thesis, Sergio Arouca National School of Public Health.

[38] Buchanan L , Kelly B & Yeatman H (2017) Exposure to digital marketing enhances young adults’ interest in energy drinks: an exploratory investigation. PLoS One 12, e0171226. 28152016 10.1371/journal.pone.0171226 PMC5289551

[39] Abid T , Abid-Dupont MA & Moulins JL (2019) What corporate social responsibility brings to brand management? The two pathways from social responsibility to brand commitment. Corp Soc Responsible Environ Manag 27, 952–936.

[40] Antúnez L , Alcaire F , Brunet G et al. (2021) COVID-washing of ultra-processed products: the content of digital marketing on Facebook during the COVID-19 pandemic in Uruguay. Public Health Nutr 24, 1142–1152. 33494846 10.1017/S1368980021000306 PMC7900666

[41] Gerritsen S , Sing F , Lin K et al. (2021) The timing, nature and extent of social media marketing by unhealthy food and drinks brands during the COVID-19 pandemic in New Zealand. Front Nutr 8, 65. 10.3389/fnut.2021.645349 PMC7973089 33748177

[42] White M , Nieto C & Barquera S (2020) Good deeds and cheap marketing: the food industry in the time of COVID-19. Obesity 28, 1578–1579. 32441869 10.1002/oby.22910 PMC7280662

[43] BBC News Brasil (2020) The routine of threats and expulsions of outsourced delivery drivers from IFood. https://www.bbc.com/portuguese/geral-53521791 (accessed July 2021).

[44] Franco DS & Ferraz DLDS (2019) Uberization of labor and capitalist accumulation. Cad EBAPE BR 17, 844–856.

[45] BBC News Brasil (2020) How delivery apps are bankrupting small restaurants. https://www.bbc.com/portuguese/geral-51272233 (accessed July 2021).

[46] World Cancer Research Fund International (2020) Building Momentum: Lessons on Implementing Robust Restrictions of Food and Non-Alcoholic Beverage Marketing to Children. https://wcrf.org/buildingmomentum (accessed August 2022).

