# Supplementary material for: Digital marketing of online food delivery services in a social media platform before and during COVID-19 pandemic in Brazil
Source: Public Health Nutr. 2022 Oct 10;26(1):1–11. doi: 10.1017/S1368980022002191 (PMC11074985; doi:10.1017/S1368980022002191)
Supplement: Supplementary file 1 [file S1368980022002191sup001.docx]

**Supplemental table S1:** Definitions of food groups and marketing strategies used to code posts, Brazil.

| **Phase 1: Nutritional quality of foods^a^** | |
| --- | --- |
| Group 1: Unprocessed or minimally processed foods or hand-prepared dishes based on these foods | Foods that have not undergone any kind of industrial processing (e.g., raw fruits and vegetables), foods that were processed in ways that did not add substances or subtract edible parts (e.g., plain yogurt), and foods that had an edible part subtracted but no substance added (e.g., pasta). Also, hand-prepared preparations or combinations in which the main component is a food that has not undergone any kind of industrial processing (e.g., fruit salad) and or that had an edible part subtracted, but no substance added (e.g., artisanal breads). |
| Group 2: Processed foods | A manufactured combination of a food with salt, oil, and/or sugar (e.g., whole fruits canned in syrup) or a handmade preparation or combination in which the main component is a food manufactured by adding salt, sugar, oils, and/or fats (e.g., handmade sandwiches from industrialized breads made without additives or synthetic substances). |
| Group 3: Ultra-processed foods | Formulations made by the food industry mostly from substances extracted from foods, such as oils, fats, starches, and sugar, and substances obtained with the further processing of foods’ constituents or through chemical synthesis, such as hydrogenated fats, and additives (e.g., confectionary, instant noodles) or handmade preparations or combinations in which the main component is a formulation made by the food industry (e.g., sandwiches made from breads made with additives or synthetic substances). |
| **Phase 2: Marketing strategies ^b,c^** | |
| Corporate social responsibility | Statement of charitable work or ethical or sustainable initiatives or stances undertaken by the brand |
| Celebrities | High-profile entertainment or media figures, excluding athletes |
| Sportspeople | Individuals who show athletic ability and/or sporting achievements, including extreme and motorsports |
| Children’s characters | Notable third-party cartoons or characters from films, books, TV, and the internet |
| Branded characters | Characters developed by the brand itself |
| Special price promotion | Reduced-price advertisements including discounts, two- for-one deals, or limited time offers |
| Vouchers | Offers only accessible to those who like the account, including print-off and/or electronic codes |
| Competitions | Contests that require participant entry and include minimum requirements such as liking a post |
| Engagement | Posts that prompt conversation or interaction |
| Meme^d^ | Content that can be viralized on the internet and modified during peer-to-peer transmission process |
| Sponsorships/partnerships | Events that the brand supports or brands/service partners – excluding charitable organizations |
| Videos/graphics interchange format (GIFs)/boomerangs | Moving images |
| Links | Links to additional content, external pages |
| Branding elements | Distinct colors, logos, fonts, slogans, or trademarks |
| Product imagery (unbranded) | Pictures of the products sold or their ingredients, with no other branding elements or labels |
| Informational content | Presence of informative content, for example, about the sensory characteristics of the product, ingredients used in the preparation, restaurant that sells it, how to use OFDS app functions |
| Original content | Original content created by the brand, instead of ‘regrammed’ images from another Instagram user where the user’s account with the original post is tagged in the post |
| Health claims | Products are portrayed as healthy choices or posts contained health claims, statements that the product can improve physical health |
| **Phase 3: COVID-19 marketing strategies ^c^** | |
| Appropriating frontline workers | Frequency with which companies attempt to link their products to the work of health professionals, emergency services, and other frontline workers during the pandemic |
| Combating the pandemic via promotions | As governments started to impose lockdown restrictions, food manufacturers and retailers faced unprecedented challenges – therefore ‘combatting the pandemic’ included: |
|  | • linking increased consumption of food with a sense of civic duty, e.g., supporting affected farmers or other workers |
|  | • COVID-19 inspired adaptations to marketing campaigns, e.g., invoking patriotic duty by encouraging consumers to do their part |
|  | • waiving delivery fees |
|  | • making use of stay-at-home orders and good hygiene practices |
| Selling social distancing | Companies have attempted to leverage opportunities associated with the distinctive social and economic context of the pandemic, with product promotions exploiting a focus on: |
|  | • social distancing |
|  | • utilizing face masks as advertising space |
|  | • inserting brand images into online video communication platforms |
| Accelerating digitalization | Companies used the changing social and economic context of lockdown to drive strategic changes in the marketing, promotion, and distribution of their products via digital means |
|  | • marketing efforts are made across digital, social, and TV platforms, for instance popular social media platforms such as ‘TikTok’ or popular games such as Nintendo®’s ‘Animal Crossing’ |
|  | • use of marketing messages encouraging people to order food for people who are physically distant or reinforcing that OFDS customers are somehow connected by using this service |
| Social responsibility in the pandemic ^d^ | Statement of or stimulus to practice any action of social responsibility, divided into: |
|  | • individual: Encouragement for people to tip the delivery personnel, to donate money to local restaurants, or to donate food or money for people in food insecurity during the pandemic |
|  | • corporate: Creation of solidarity or protection funds for delivery personnel, distribution of personal protective equipment (PPE) for delivery personnel, charity targeted to people in vulnerability due to the pandemic. |
| Support for delivery personnel ^d^ | In addition to social responsibility actions, expressions of appreciation for delivery work, highlighting its importance during the pandemic and encouragement for people to do the same. |
| Support for restaurants ^d^ | In addition to social responsibility actions, advertising local restaurants open for delivery, encouragement for people to cite their favorite restaurants ant to buy from local ones during the pandemic. |
|  |  |

^a^Louzada et al., 2015. ^b^Vassalo et al., 2018. ^c^Jia et al., 2021. ^d^Variables that emerged from content analysis.

**Supplemental table S2: COVID-19 Marketing Strategies – Case Studies**

| Note: all images are publicly available on the respective Instagram account via the URL. | | |
| --- | --- | --- |
| **Strategy** | **Figure 1** | **Figure 2** |
| ‘social responsibility during the pandemic’ - 'corporate social responsibility’ | 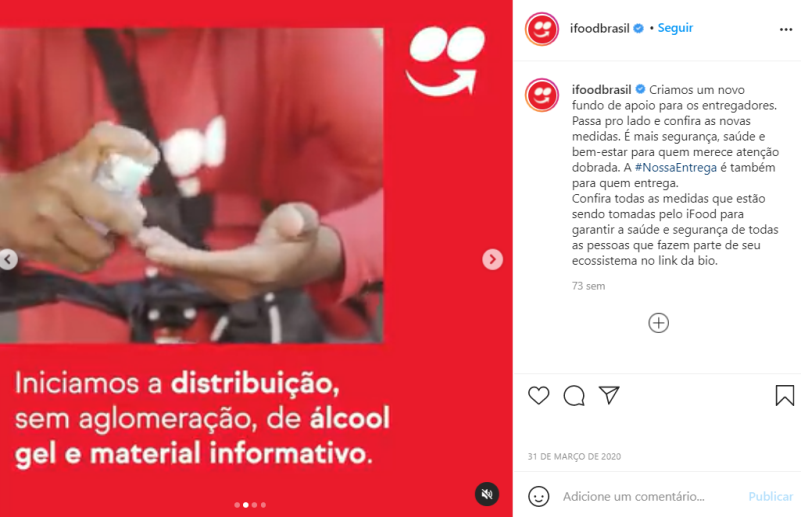  <https://www.instagram.com/p/B-ZgbEgJeEX/>  Fig. 1 Post by iFood® to announce the creation of a fund to delivery drivers and distribution of informational materials on COVID-19 and hand sanitizers to them. The text reads: “Check out on the bio link all the measures that are being taken by iFood to ensure the health and safety of all people who are part of its ecosystem.” | 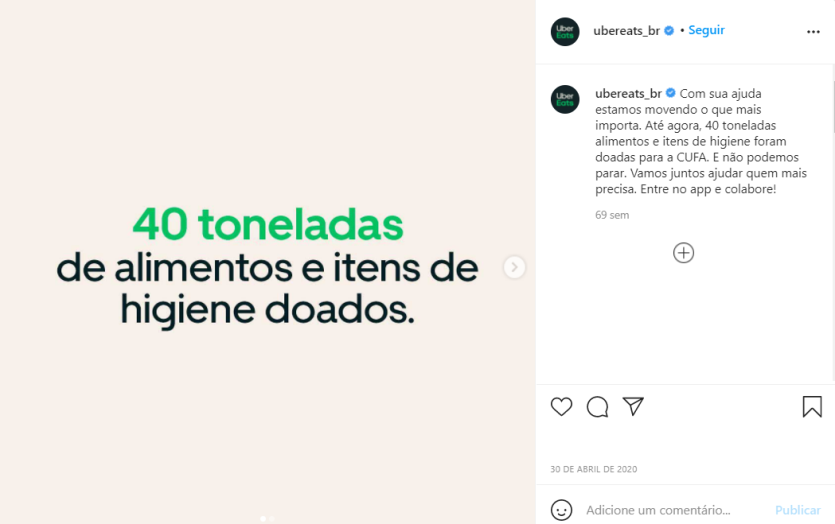  <https://www.instagram.com/p/B_noGyJF6mn/>  Fig. 2 Post by UberEats® to announce the donation of 40 tons of food and hygiene products to a nongovernmental organization. The text reads: “So far, 40 tons of food and hygiene items have been donated to CUFA. And we can't stop. Let's join together to help those most in need. Enter the app and collaborate!”. CUFA or Central Única das Favelas is a Brazilian non-governmental organization that promotes education, leisure, sports, culture, and citizenship activities in low-income communities. |
|  | **Figure 3** | **Figure 4** |
| ‘social responsibility during the pandemic’ - ‘individual social responsibility’ | 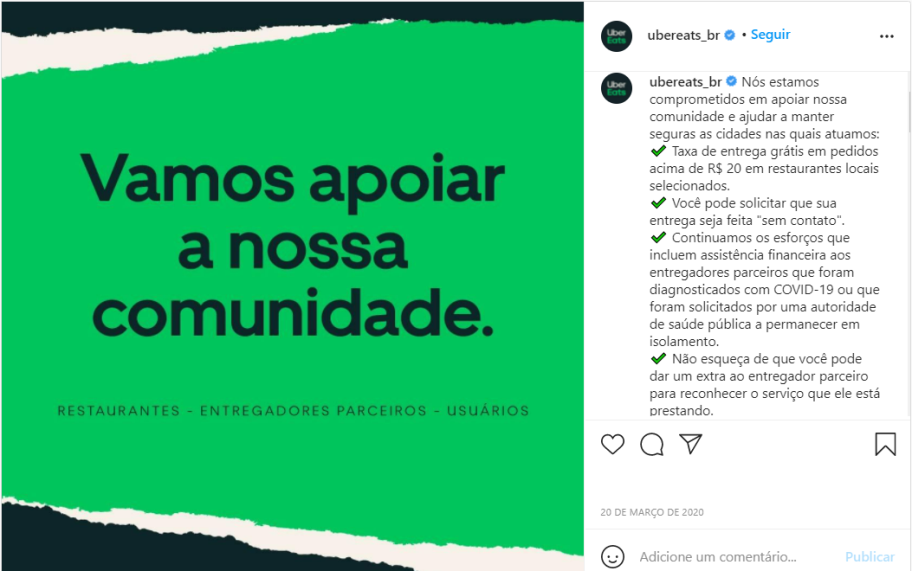  <https://www.instagram.com/p/B9-MHA_FbOt/>  Fig. 3 Post by UberEats® to announce its commitment to support communities and keep cities safe. The text reads: “We are committed to supporting our community and helping keep the cities we serve safe: Free delivery fee on orders over R$20 [equivalent to US$ 3,6] at selected local restaurants. You can request that your delivery be made "contactless". We continue efforts that include financial assistance to partner delivery drivers who have been diagnosed with COVID-19 or who have been requested by a public health authority to remain in isolation. Don't forget that you can give extra money to the partner delivery driver to thank them for the service they are providing.”. | 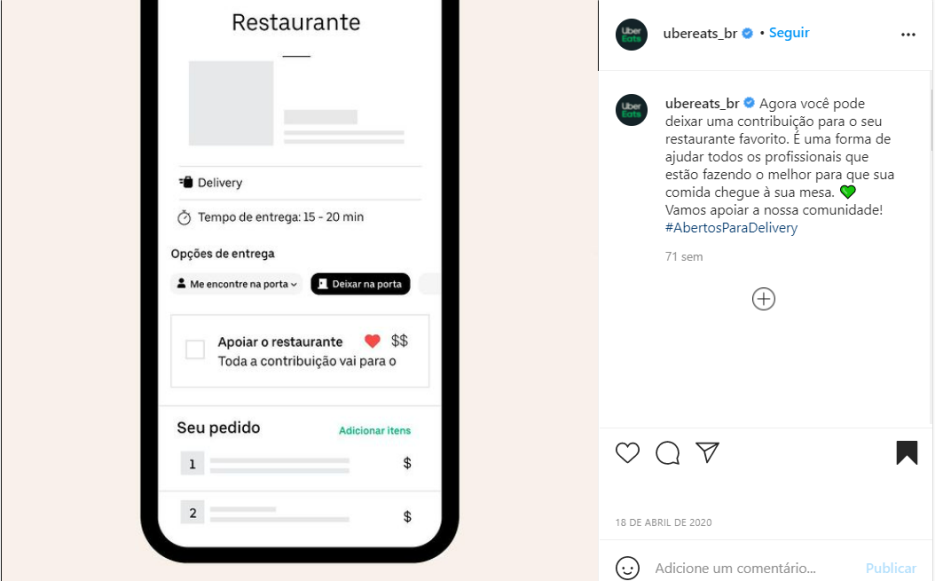  <https://www.instagram.com/p/B_IF8-vllrR/>  Fig. 4 Post by UberEats® to announce the new possibility in the app to contribute to the restaurant when ordering. The text reads “Now you can contribute to your favourite restaurant. It's a way to help all professionals who are doing their best to get food to your table.”. |
|  | **Figure 5** | |
| ‘social responsibility during the pandemic’ - ‘individual social responsibility’ (continuation) | 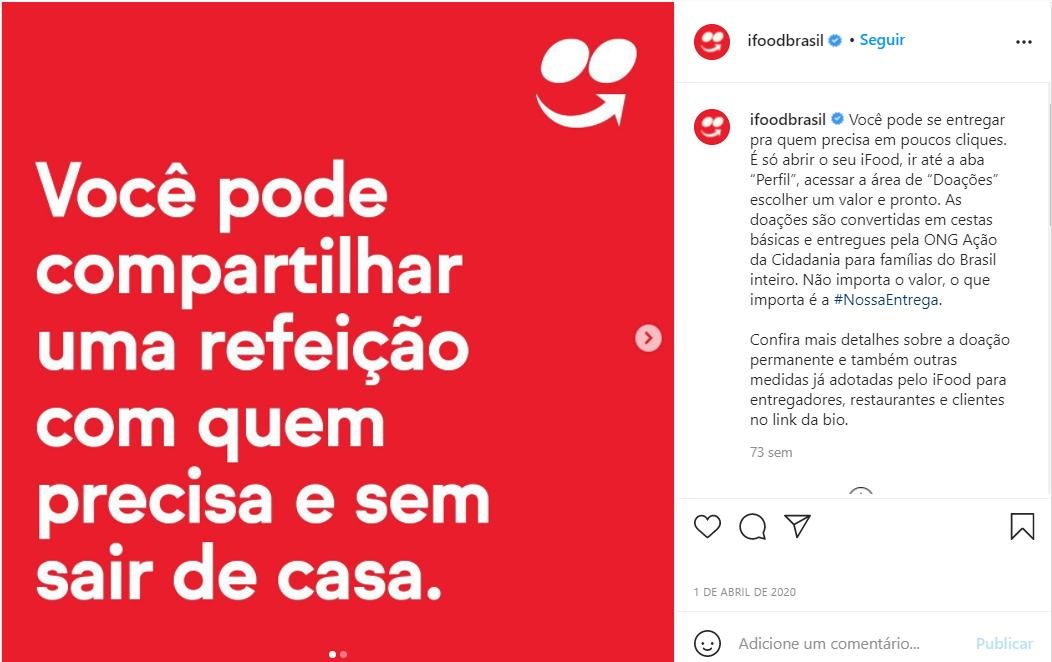 <https://www.instagram.com/p/B-dJR2eJh5c/>  Fig. 5 Post by Ifood® explaining how to donate to nongovernmental organizations via its platform. The image says: “You can share a meal with whoever needs it without leaving your home.” The text reads “Just open iFood, go to the "Profile" tab, access the "Donations" area, choose an amount and that's it. Donations are converted into food baskets and delivered by the NGO Ação da Cidadania to families throughout Brazil.” and “Check out the bio link for more details on permanent donations and other measures by iFood to help delivery personnel, restaurants, and customers.” | |
|  | **Figure 6** | |
| ‘combatting the pandemic’ | 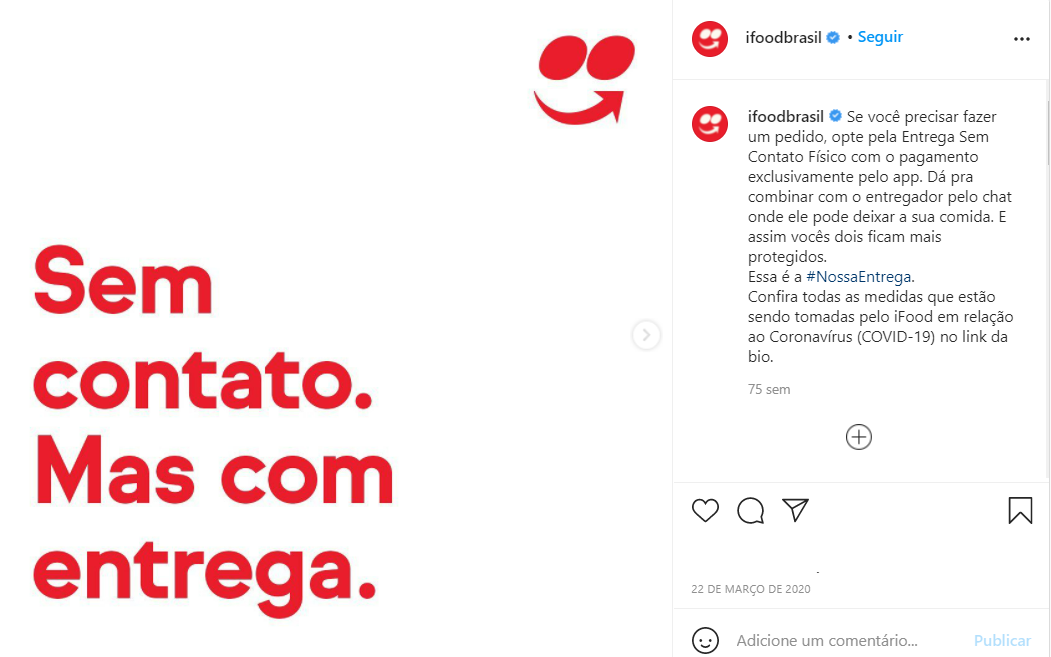<https://www.instagram.com/p/B-DP4ztp2AD/>  Fig. 6 Post by iFood® to announce contactless delivery, explaining “You can arrange with the delivery driver through the chat where he can leave your food. So, you´re both more protected.” | |
|  | **Figure 7** | **Figure 8** |
| Accelerating digitalization | 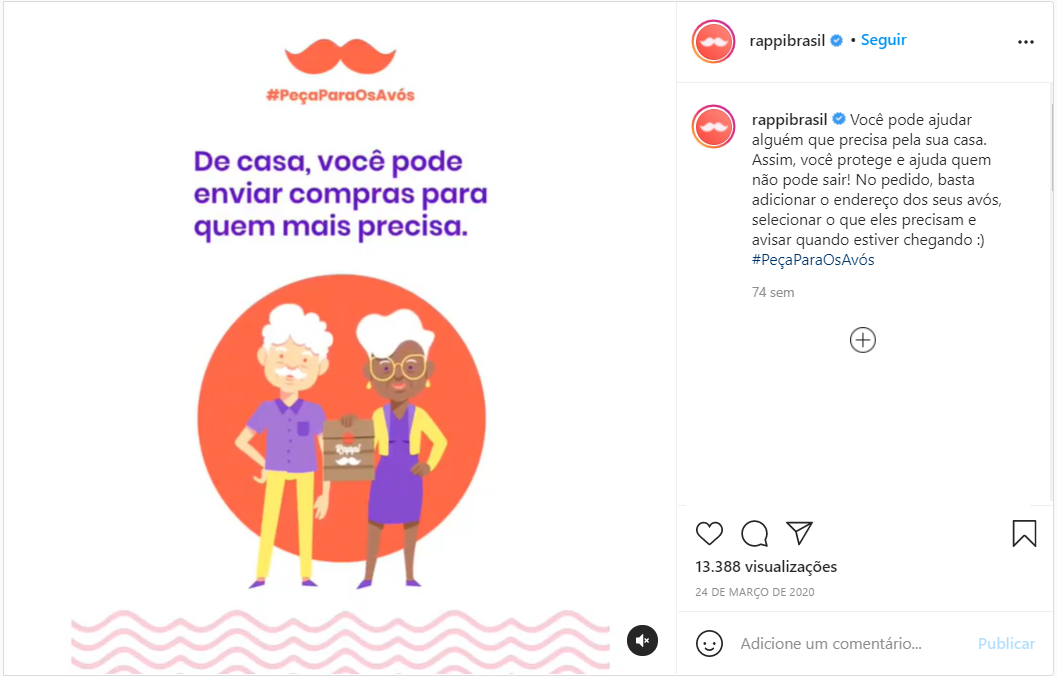  <https://www.instagram.com/p/B-IRYQ7po8N/>  Fig. 7 Post by Rappi® to announce delivery orders from one consumer to another using the platform. The text reads: “You can help someone in need from your own home. You protect and help those who can´t leave! When ordering, just add your grandparents' address, select what they need, and let them know when the food is arriving.” | [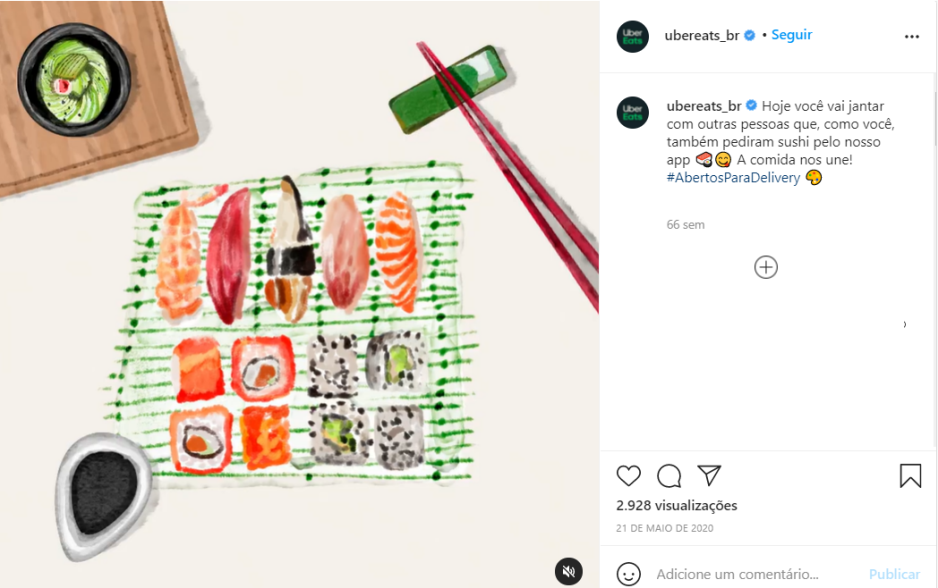](https://www.instagram.com/p/CAyaKz4F5fq/)  <https://www.instagram.com/p/CAyaKz4F5fq/>  Fig. 8 Post by UberEats® with the slogan “Food unites us” using sushi as an example. The text reads: “Many people are eating the same thing as you for dinner. You keep each other company. Food unites us.”. |

\

|  | **Figure 9** | **Figure 10** |
| --- | --- | --- |
| Accelerating digitalization (continued) | 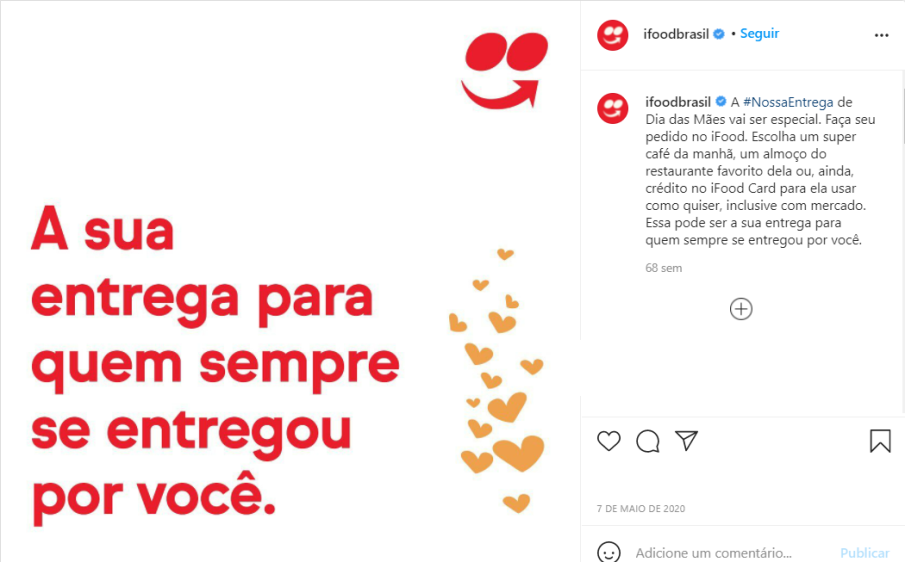  <https://www.instagram.com/p/B_5n0FIp5q4/>  Fig. 9 Post by iFood® suggesting use of the platform on Mother’s Day to “Choose a super-breakfast, lunch at her [the reader´s mother´s] favorite restaurant, or even an iFood Gift Card for her to use as she pleases, including at a grocery store.” | 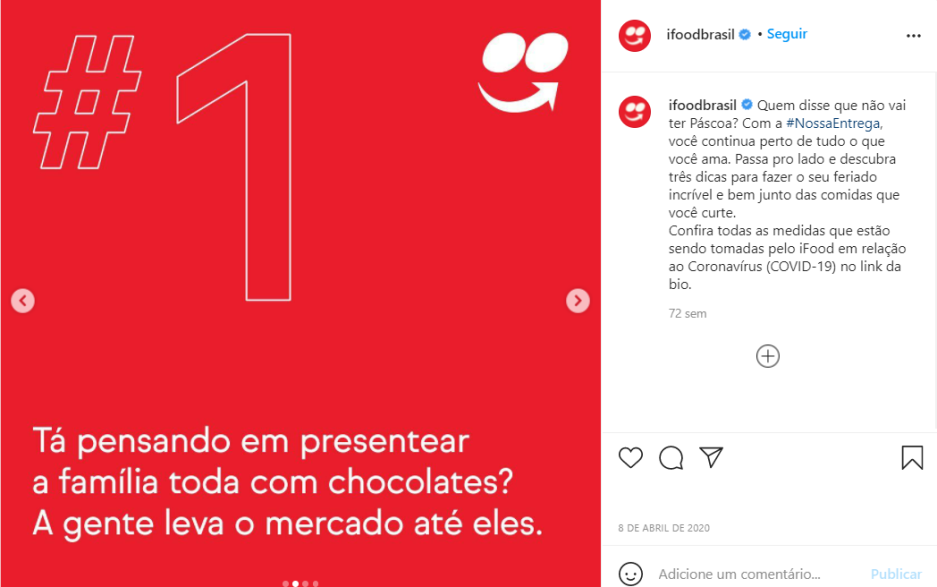  <https://www.instagram.com/p/B-u7rofpmk5/>  Fig. 10 Post by iFood® to encourage use of the platform on Easter. Text reads: “Who said there won't be Easter? With #OurDeliver you stay close to everything you love.” In the third image of the post, the text reads “To keep everyone you care about safe, count on us to deliver all your Easter items with Contactless Delivery.”. |

|  | **Figure 11** | **Figure 12** |
| --- | --- | --- |
| ‘support for delivery drivers’ | 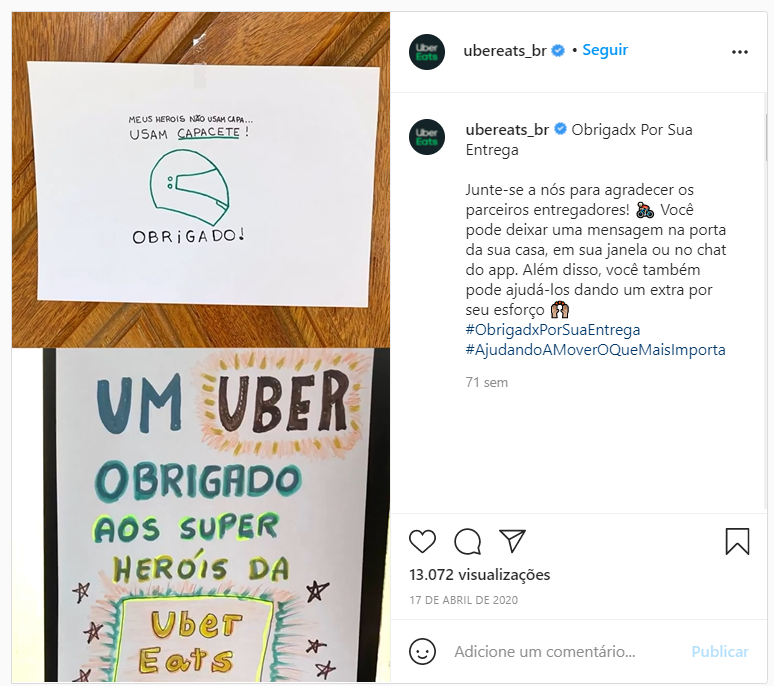  <https://www.instagram.com/p/B_FryI9Fuqr/>  Fig. 11 Print from a video post by UberEats® showing drawings with messages left by customers to thank delivery drivers. The text reads: “Join us to thank our delivery partners! You can leave a message on your doorstep, in your window, or in the app's chat. You can also help them by tipping extra for their efforts.” | 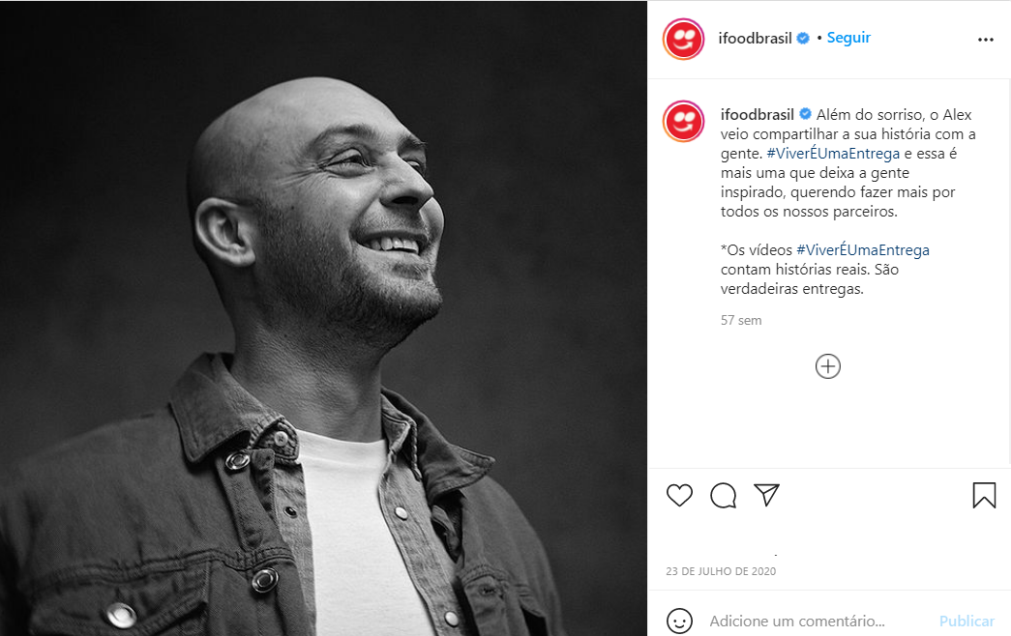  <https://www.instagram.com/p/CC_bH31pOnS/>  Fig. 12 Post by iFood® telling a delivery driver´s story. The text reads that his story “is another one that inspires us to do more for all our partners.” |

|  | **Figure 13** | **Figure 14** | |
| --- | --- | --- | --- |
| ‘support for restaurants’ | 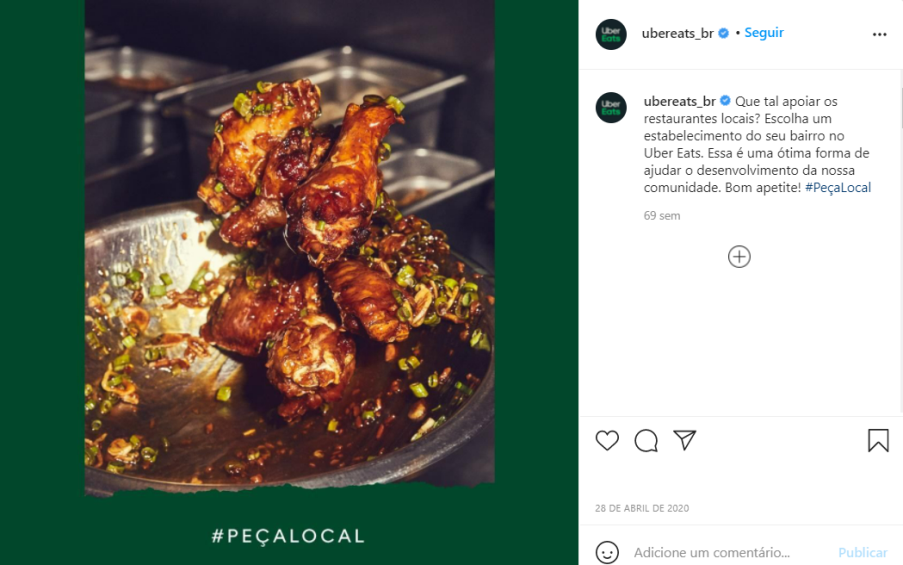  <https://www.instagram.com/p/B_iFWeal-Vc/>  Fig. 13 Post by UberEats® to encourage support for local restaurants. The text reads: “How about supporting local restaurants? Choose an establishment in your neighborhood at Uber Eats. This is a great way to help our community develop. Enjoy your food!” | 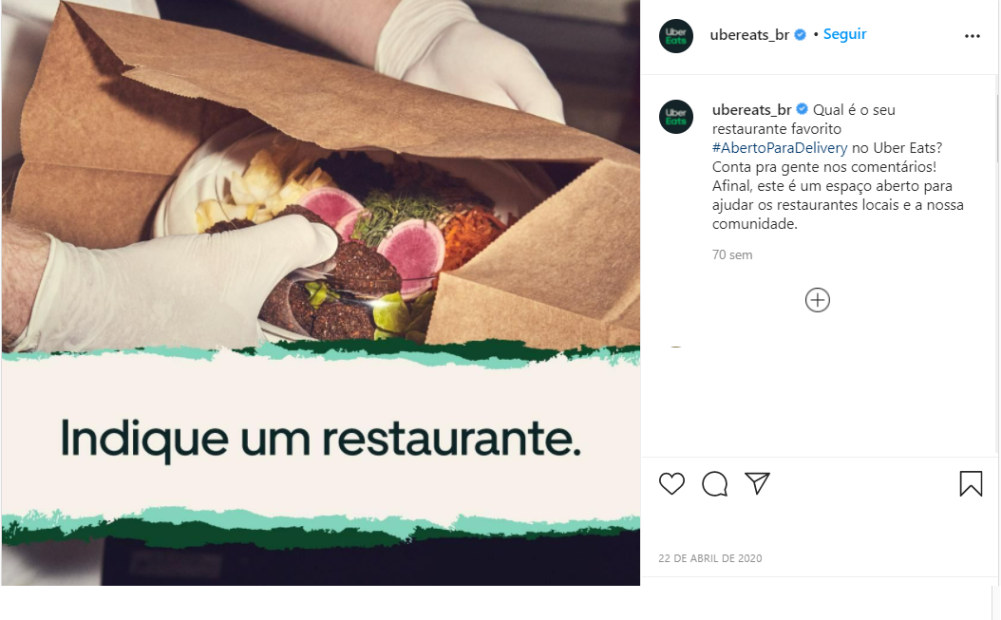  <https://www.instagram.com/p/B_S4BKjAAhN/>  Fig. 13 Post by UberEats® to foster public interaction to write comments on their favorite restaurants on the platform. The text reads: “What is your favorite #OpenForDelivery restaurant at Uber Eats? Tell us in the comments! After all, this is an open space to support local restaurants and our community.” | |
|  | **Figure 15** | |  |
| ‘support for restaurants’ (continued) | 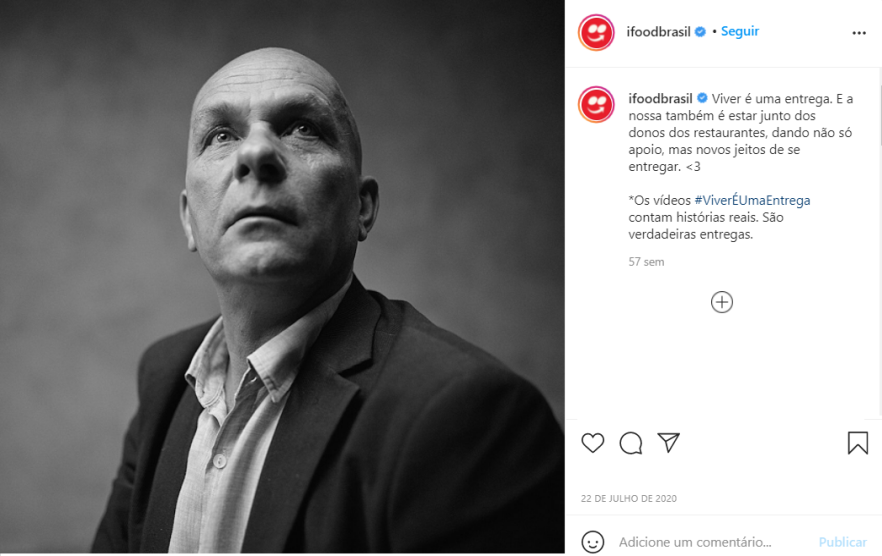<https://www.instagram.com/p/CC8o0LtpIXO/>  Fig. 15 Post by iFood® to highlight that the platform is “with the restaurant owners” and to tell a restaurant owner´s real-life story. | |  |
